# New insight into the molecular mechanism of miR482/2118 during plant resistance to pathogens

**DOI:** 10.3389/fpls.2022.1026762

**Published:** 2022-10-28

**Authors:** Lijuan Liao, Biao Xie, Peipei Guan, Ning Jiang, Jun Cui

**Affiliations:** ^1^ College of Life Science, Hunan Normal University, Changsha, China; ^2^ Hunan Provincial Key Laboratory for Microbial Molecular Biology, Changsha, China; ^3^ The National & Local Joint Engineering Laboratory of Animal Peptide Drug Development, Changsha, China; ^4^ Hunan Academy of Agricultural Sciences, Changsha, China

**Keywords:** MIR482/2118, miR482/2118-3p/5p, phasiRNA, lncRNA, resistance

## Abstract

MicroRNAs (miRNAs), a group of small noncoding RNAs (approximately 20-24 nucleotides), act as essential regulators affecting endogenous gene expression in plants. MiR482/2118 is a unique miRNA superfamily in plants and represses *NUCLEOTIDE BINDING SITE-LEUCINE-RICH REPEAT* (*NBS-LRR*) genes to function in plant resistance to pathogens. In addition, over the past several years, it has been found that miR482/2118 not only targets *NBS-LRR*s but also acts on other molecular mechanisms to affect plant resistance. miR482/2118-5ps, phased small interfering RNAs (phasiRNAs) and long noncoding RNAs (lncRNAs) play important roles in plant disease resistance. This review summarizes the current knowledge of the interactions and links between miR482/2118 and its new interacting molecules, miR482/2118-5p, phasiRNAs and lncRNAs, in plant disease resistance. Here, we aim to provide a comprehensive view describing the new molecular mechanism associated with miR482/2118 in the plant immune system.

## Introduction

**Figure 1:**
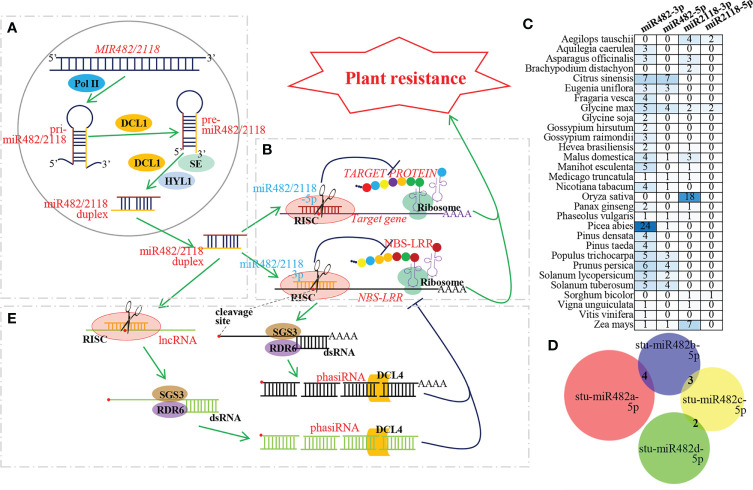
Biogenesis and mechanism of miR481/2118 superfamily during plant resistance to pathogen. **(A)** Biogenesis pathway of miR482/2118. MIR482/2118 genes are transcribed by Pol II into pri-miR482/2118. Then, pri-miR482/2118 of hairpin-like structure produces pre-miR482/2118 by DCL1. By DCL1, HYL1 and SE, pre-miR482/2118 is then processed into a miR482/2118-3p/miR482/2118-5p duplex. The mature miR482/2118-3p or miR482/2118-5p liberated from the duplex is commonly incorporated into RISCs to their targets to mediate gene silencing. **(B)** MiR482/2118-3p and -5p co-regulating plant resistance. MiR482/2118-3p and -5p cleave their target genes to inhibit translation of resistant protein, thus, regulating plant resistance. **(C)** Quantity variation of miR482/2118-3p and -5p in different plant species. MiRNA resources are downloaded from miRBase database (https://www.mirbase.org/). The numbers in the heat map represent the number of miRNAs. **(D)** Number of the identical target genes among *Solanum tuberosum* miR482s (stu-miR482). The target genes are predicted by psRNAtarget (https://www.zhaolab.org/psRNATarget/) with Expectation ≤ 4. Venn diagram is performed by DeepVenn (http://www.deepvenn.com/). **(E)** PhasiRNA biogenesis and function during plant resistance to pathogen. 3’ fragment of cleaved target gene or lncRNA are converted by RDR6 and SGS3 into double-stranded RNAs, and then they are processed into phasiRNAs through continuous DCL4 chopping.

MicroRNAs (miRNAs), approximately 22 nucleotides (nt) of endogenous small noncoding RNA, have been discovered to act as master regulators affecting endogenous gene expression in plants (Reinhart et al., 2002; Rhoades et al., 2002). In plant miRNAs, miR482/2118 is a unique miRNA superfamily consisting of two mature miRNA isoforms, miR482 and miR2118 (both 22 nt in length) (Shen et al., 2020). For miR482/2118 biogenesis, first, MIR482/2118 genes are transcribed by RNA polymerase II (Pol II) into long primary miR482/2118 (pri-miR482/2118). pri-miR482/2118 are polyadenylated and stranded RNA molecules that fold into hairpin-like structures and produce precursors of miR482/2118 (pre-miR482/2118) by the RNase III family enzyme DICER-LIKE1 (DCL1). By DCL1, HYPONASTIC LEAVES 1 (HYL1) and Serrate (SE), pre-miR482/2118 is then processed into a miR482/2118 duplex consisting of miR482/2118 and complementary miR482/2118*. MiR482/2118 strand is called the guide strand, and miR482/2118* strand is called the passenger strand. The liberated strands have also been defined as miR482/2118-3ps and miR482/2118-5ps, according to the 5’ and 3’arms of the hairpin precursor, after renaming by the miRBase registry. Once liberated from the duplex, mature miR482/2118-3p or miR482/2118-5p is commonly incorporated into specific ARGONAUTE (AGO)-associated RNA-induced silencing complexes (RISCs) and guides RISCs to their targets to mediate gene silencing (Xiao and Luan, 2014; Liu et al., 2017; Zhang et al., 2022) (**Figure 1A, B**).

The plant immune system, a ‘zig-zag-zig’ model, is composed of two layers of defense responses that provide protection against pathogens, including pathogen-associated molecular pattern (PAMP)-triggered immunity (PTI) and effector-triggered immunity (ETI) (Jones and Dangl, 2006; Fei et al., 2016). PTI is the first layer of defense. Membrane-localized pattern recognition receptors (PRRs) function in the recognition of PAMPs to cause plant immune responses, such as stomatal closure and a burst of reactive oxygen species. ETI acts more strongly in its amplitude of defense. ETI is activated by nucleotide-binding domain leucine-rich repeat containing receptors (NLRs) recognizing effectors secreted by pathogens, leading to hypersensitive response (HR). PTI and ETI do not function independently but interdependent and mutually reinforcing. The production of reactive oxygen species by the NADPH oxidase RBOHD is a critical early signalling event connecting PTI and ETI, and the potentiation of PTI is an indispensable component of ETI during pathogen infection (Yuan et al., 2021). moreover, in *Oryza sativa*, the deubiquitinase PICI1 is identified as an immunity hub for PTI and ETI. PICI1 is targeted for degradation by blast fungal effectors to dampen PTI. NLRs protect PICI1 from effector-mediated degradation to reboot the methionine-ethylene cascade (Zhai et al., 2022). Nucleotide-binding site leucine-rich repeat (NBS-LRR) resistance proteins are important members of NLR family. MiR482/2118 members target conserved sequences encoding the P-loop of *NBS-LRR* genes, thus inhibiting the expression of *NBS-LRR* genes (Jiang et al., 2018b). This suggests that the miR482/2118-NBS-LRR module is involved in the ETI.

Many previous studies have shown that the miR482/2118-NBS-LRR module acts in the resistance of various plants to pathogens. For example, the increased susceptibility of stu-miR482e-overexpressing potato plants to *Verticillium dahliae* infection can be explained by the enhancement of stu-miR482e-mediated silencing of NBS-LRR disease-resistance genes (Yang et al., 2015). During infection with *Phytophthora infestans* and *Botrytis cinerea*, sly-miR482b inhibits the expression of its target genes, NBS-LRRs, and transgenic tomato and *Arabidopsis* overexpressing sly-miR482b show decreased resistance (Jiang et al., 2018a; Wu et al., 2021). In addition, over the past several years, miRNA-5p, phased small interfering RNAs (phasiRNAs) and long noncoding RNAs (lncRNAs) have been involved in plant resistance to pathogens (Xia et al., 2015; Cui et al., 2017; Liu et al., 2017). pre-miR482/2118 can produce miR482/2118-5p; miR482/2118s target the transcripts to trigger phasiRNA production; lncRNAs inhibit miR482/2188s expression, and miR482/2188s target lncRNAs (Canto-pastor et al., 2019; Jiang et al., 2019; Jiang et al., 2020; Liu et al., 2022a). Here, we aim to provide a comprehensive view describing the possible integration of miR482/2118-5p, phasiRNAs and lncRNAs into the plant immune system associated with miR482/2118.

## MiR482/2118-3p and -5p co-regulateplant resistance

MiRNA-5p was originally thought to be a nonfunctional and degradable byproduct formed during miRNA biogenesis, since the accumulation of miRNA-5p is much lower than that of miRNA-3p in plants (Liu et al., 2017). However, an increasing number of studies have demonstrated that miRNA-5p, as a regulatory factor, plays important roles in a variety of other biological processes, including plant resistance to pathogens (Zhang et al., 2011; Nie et al., 2019).

The miRBase database (https://www.mirbase.org/) contains 191 members of the miR482/2118 superfamily from 30 plant species, including 153 miR482/2118-3ps and 38 miR482/2118-5ps (**Figure 1C**). Previous studies have shown that the target genes of miR482/2118-3ps are mainly members of the NBS-LRR family (Jiang et al., 2018b). In contrast, the target genes of miR482/2118-5p superfamily members vary due to their short conserved sequence. There are few identical target genes among members of the same genus. For example, *Solanum tuberosum* contains four members of the stu-miR482/2118-5p superfamily, stu-miR482a/b/c/d-5p. There were four identical target genes between stu-miR482a-5p and stu-miR482b-5p, three between stu-miR482b-5p and stu-miR482c-5p, and two between stu-miR482c-5p and stu-miR482d-5p, but there were no identical target genes among all four members due to the diversification of stu-miR482 family and the diversification of stu-miR482 family and the nucleotide diversity of the P-loop motif of *NBS-LRRs*, especially the wobble position of the codons in the target site of miR482/2118 (Zhang et al., 2022) (**Figure 1D**).

Tomato miR482d-3p/-5p and miR482e-3p/-5p can respond to pathogen infection. Sly-miR482e-5p is significantly downregulated in both Moneymaker tomato (susceptible cultivar to *Fusarium oxysporum*) and Motelle tomato (resistant cultivar to *F. oxysporum*) upon *F. oxysporum* infection. Levels of sly-miR482e-3p and sly-miR482d-3p are suppressed in Motelle but increased in Moneymaker after *F. oxysporum* treatment. However, sly-miR482d-5p presents the opposite pattern, showing decreased levels in Moneymaker and increased amounts in Motelle after infection with *F. oxysporum* (Ji et al., 2018). After infection with P. infestans, both sly-miR482e-3p and -5p are downregulated in Zaofen No. 2 tomato, but the expression level of sly-miR482e-5p is even higher than that of sly-miR482e-3p. Transgenic plants that overexpressed sly-miR482e-3p or -5p show susceptibility to the pathogen, lower expression levels of PR1 and PR5 genes, more number of necrotic cells and the reactive oxygen species (ROS) accumulation, while these results are reversed after miR482e-3p or miR482e-5p silencing. These results suggest that sly-miR482e-3p and -5p induces PR gene expression and reduces the ROS accumulation to protect against cell membrane injury, leading to enhanced resistance to *P. infestans.* (Liu et al., 2022a; Liu et al., 2022b).

These results suggest that miR482/2118-3p and -5p coregulate plant resistance.

## MiR482/2118 mediates phasiRNA generation

In addition to targeting genes, it has been demonstrated that some miRNA-mediated cleavages of transcripts can trigger the production of phasiRNA (Gai et al., 2018; Bélanger et al., 2020; López-Márquez et al., 2021; Shi et al., 2022). PhasiRNAs, another major class of small RNAs in plants, are involved in the control of plant biological processes (Guo et al., 2018). The biogenesis of phasiRNAs occurs after cleavage of the targets by miRNA. After cleavage, the 3’fragment is converted to dsRNA *via* the activity of RNA-DEPENDENT RNA POLYMERASE6 (RDR6), assisting by SUPPRESSOR OF GENE SILENCING3 (SGS3). The resulting dsRNA is iteratively cleaved by a member of Dicer protein family, such as DCL4, from the 5’ end of strand containing the cleavage site, yielding duplexes of phasiRNAs (Liu et al., 2020; Zhang et al., 2022). These phasiRNAs can, in turn, *cis*-cleave their precursor or *trans*-regulate other target genes simultaneously (Liu et al., 2020) (**Figure 1E**). There are two pathways for the biogenesis of phasiRNAs, named “one-hit” and “two-hit” pathways. The one-hit pathway is typified by a single target site for a 22-nucleotide miRNA that results in downstream processing of the target transcript into 21-nucleotide phasiRNAs, while the two-hit pathway is typified by two target sites of a 21-nucleotide miRNA that results in processing upstream of the 3’ site. (Fei et al., 2013; Liu et al., 2020).

In eudicots, miR482/2118 superfamily members can target a number of *NBS-LRRs* and trigger phasiRNA production, and the resulting phasiRNAs can play important roles in enhancing the silencing effects of miR482/2118 on *NBS-LRRs* (Canto-Pastor et al., 2019; Shivaprasad et al., 2012; Zhai et al., 2011). As observed in several eudicots, *NBS-LRR* genes comprise the largest class of genes producing phasiRNAs (PHAS genes) in spruce, and miR482/miR2118, encoded in spruce by at least 24 precursor loci, targets *NBS-LRR* genes to trigger phasiRNA production (Xia et al., 2015) (**Figure 1E**).

In addition, miR482/2118 superfamily members can cleave lncRNAs to trigger the generation of phasiRNAs. Spruce miR482/2118 targets noncoding PHAS loci to trigger phasiRNA production, with the latter enriched in reproductive tissues (Xia et al., 2015). Similarly, in litchi, miR482 directs phasiRNA generation from long noncoding genes *via* alternative splicing ions from long noncoding genes *via* alternative splicing (AS) and alternative polyadenylation (APA) (Ma et al., 2018). A lncRNA formed by a rearrangement of several CNLs and TNLs, named TAS5, was identified in tomato. TAS5 is targeted by miR2118b, triggering the generation of phasiRNAs that act in *trans* to regulate multiple NBS-LRRs (Canto-Pastor et al., 2019; Zhang et al., 2022) (**Figure 1E**).

These results suggest that phasiRNAs triggered by members of the miR482/2118 superfamily may act in regulating plant resistance by affecting NBS-LRR levels.

## CeRNAs inhibits MIR482/2118 expression

Competitive endogenous RNAs (ceRNAs) were first proposed by Salmena (Salmena et al., 2011). Many research papers suggest that lncRNAs can act as ceRNAs through competitive binding of miRNAs, releasing or attenuating repression by sequestering miRNAs away from target mRNAs. The binding sites of miRNAs among these lncRNAs are called endogenous target mimics (eTMs). Previous studies have shown that lncRNAs act in the ‘zig-zag-zig’ model of the plant immune system by decoying miR482/2118s, enhancing target gene *NBS-LRR* levels (an essential component of ETI) (Jiang et al., 2019; Liu et al., 2022b).

**Figure 2:**
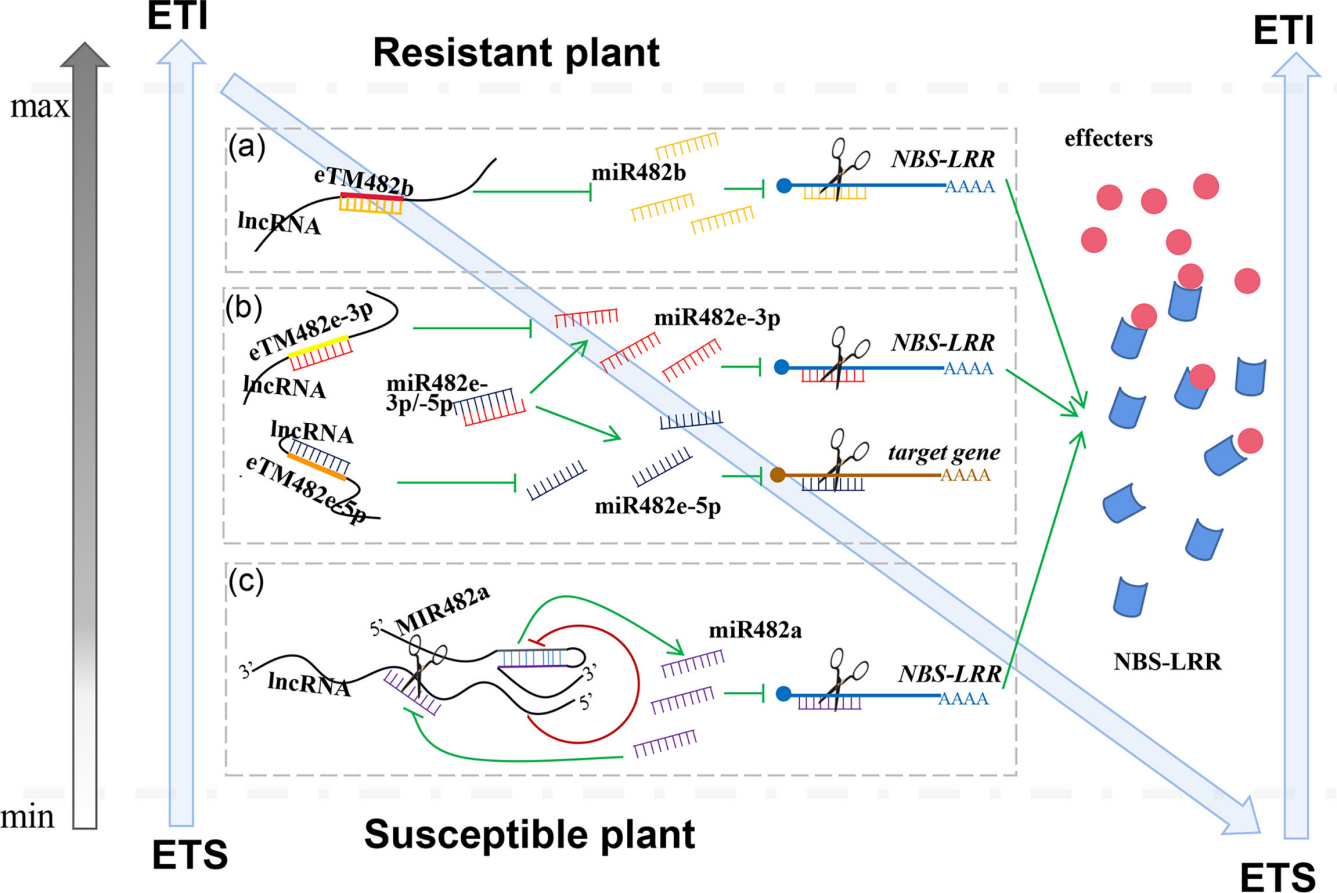
miR482/2118 and lncRNAs are important components in the effector-triggered immunity (ETI) for the plant immune response. In this extended model, lncRNAs function as ceRNAs to modulate resistant genes by decoying miR482b-3p **(A)**, miR482e-3p and -5p **(B)** in ETI for the plant innate immune response. Other lncRNAs as NATs suppress the levels of pre-miR482a to affect the expression of mature miR482a, which leads to accumulation of resistant genes, NBS-LRRs is increased **(C)**. When excessive amounts of resistant genes accumulate, mature miR482a cleaves NATs to relieve the suppression of pre-miR482a, leading to an increase in accumulation of mature miR482a and decreased accumulation of resistant genes, thus maintaining resistant genes homeostasis.

Jiang and her colleagues provide the predicted rules of ceRNAs and identified many lncRNAs as ceRNAs that play important roles in tomato resistance to *P. infestans* (Jiang et al., 2019; Cui et al., 2020). Three tomato lncRNAs, lncRNA23468, lncRNA01308 and lncRNA13262, contain conserved eTM sites for sly-miR482b. When lncRNA23468 is overexpressed in tomato, sly-miR482b expression is significantly decreased, and the expression of the *NBS-LRR* target genes is significantly increased, resulting in enhanced resistance to *P. infestans*. Silencing lncRNA23468 in tomato leads to the increased accumulation of sly-miR482b and decreased accumulation of NBS-LRRs, as well as reduced resistance to *P. infestans* (Jiang et al., 2019) (**Figure 2A**). Meanwhile, tomato lncRNA08489 acts as a ceRNA of sly-miR482e-3p to suppress sly-miR482e-3p expression and increase the expression level of its target gene *NBS-LRR* (Liu et al., 2022b) (**Figure 2**). Four lncRNAs (MSTRG.2115, MSTRG.30601, MSTRG.30599 and MSTRG.31962) respond to root−knot nematodes by acting as a decoy of a member of the miR482/2118 superfamily, gma-MIR482c-p5_2ss12GA19CT, in peanut (Xu et al., 2022) (**Figure 2B**).

In addition to silencing miR482-3p as a “sponge”, lncRNAs also repress miR482-5p expression. Due to the eTM site for sly-miR482e-5p in lncRNA39298, lncRNA39298 decoys sly-miR482e-5p to inhibit its expression, resulting in increased plant resistance to P. infestans (Liu et al., 2022a) (**Figure 2B**).

Taken together, these findings suggest that lncRNAs act in the plant immune system by decoying miR482/2118, regulating plant resistance.

### *MIR482/2118* gene expression affected by lncRNA

Another interaction mechanism between lncRNAs and miR482/2118 was also found. Tomato *MIR482a* was determined to be located on chromosome 3 in the tomato genome. After further analysis of this sequence, it was found that lncRNA15492 is located on the antisense sequence of *MIR482a* as a natural antisense transcript (NAT). Gain- and loss-of-function experiments revealed that lncRNA15492 suppressed the expression of *MIR482a* to regulate mature miR482a levels. Thus, once the expression of mature sly-miR482a was suppressed, the accumulation of NBS-LRR was increased, and tomato resistance was also enhanced. Interestingly, mature sly-miR482a can also target lncRNA15492. When excessive amounts of NBS-LRR accumulate, mature miR482a cleaves lncRNA15492 to relieve the suppression of MIR482a, leading to an increase in the accumulation of mature sly-miR482a and a decrease in the accumulation of NBS-LRR, thus maintaining NBS-LRR homeostasis (Jiang et al., 2020) (**Figure 2C**). This finding of maintaining NBS balance in plants during infection with pathogens is very significant. However, at present, this regulatory mechanism is only found in tomato MIR482a, not in all other MIR482/2118 superfamily members. The same mechanism was found in a study of *Arabidopsis* ath-miR398 function. NATs, NAT398b and NAT398c are located on the antisense sequences of MIR398b and MIR398c, respectively. Knock down of NAT398b and NAT398c upregulates MIR398b and MIR398c; overexpression of NAT398b and NAT398c represses the processing of ath-miR398 (Li et al., 2020).

## Conclusions and future perspectives

This review expands our knowledge about the intertwined regulatory role of miR482/2118 in plant resistance to pathogens. New molecules interacting with miR482/2118-3p, including miR482/2118-5p, phasiRNAs, and lncRNAs, and their regulatory mechanisms participating in plant resistance to pathogens were summarized and described in this review. Some research has shown that miR482/2118-3p and -5p coregulate plant resistance, and phasiRNAs triggered by miR482/2118 superfamily members act in regulating plant resistance by affecting NBS-LRR levels. LncRNAs not only act as ceRNAs to silence miR482/2118 but also affect *MIR482a* located on its antisense sequence during plant resistance to pathogens. However, many important issues remain to be answered, such as whether all mechanisms work together during plant resistance to pathogens and how they work; whether these disease resistance mechanisms respond to the infection of all pathogens or a certain pathogen and whether there are specific proteins or other chemical tags involved in these plant resistance mechanisms associated with miR482/2118. Overall, further research is needed to address these issues and to better understand the resistance pathway associated with miR482/2118. We believe that future studies on resistance mechanisms will provide additional insight into plant immunity and offer effective approaches for the improvement of plant disease resistance.

## Author contributions

LL and BX wrote the manuscript. LL, PG and JC collected data. JC and NJ contributed in revising manuscript. All authors contributed to the article and approved the submitted version.

## Funding

This research was supported by grants from the Natural Science Foundation of Hunan Province (No.2021JJ30441).

## Conflict of interest

The authors declare that the research was conducted in the absence of any commercial or financial relationships that could be construed as a potential conflict of interest.

## Publisher’s note

All claims expressed in this article are solely those of the authors and do not necessarily represent those of their affiliated organizations, or those of the publisher, the editors and the reviewers. Any product that may be evaluated in this article, or claim that may be made by its manufacturer, is not guaranteed or endorsed by the publisher.
